# Predicting System Degradation with a Guided Neural Network Approach

**DOI:** 10.3390/s23146346

**Published:** 2023-07-12

**Authors:** Hamidreza Habibollahi Najaf Abadi, Mohammad Modarres

**Affiliations:** Center for Risk and Reliability, Department of Mechanical Engineering, University of Maryland, College Park, MD 20742, USA; modarres@umd.edu

**Keywords:** neural network, degradation behavior, lifetime prediction, physics of degradation

## Abstract

Evaluating the physical degradation behavior and estimating the lifetime of engineering systems and structures is crucial to ensure their safe and reliable operation. However, measuring lifetime through actual operating conditions can be a difficult and slow process. While valuable and quick in measuring lifetimes, accelerated life testing is often oversimplified and does not provide accurate simulations of the exact operating environment. This paper proposes a data-driven framework for time-efficient modeling of field degradation using sensor measurements from short-term actual operating conditions degradation tests. The framework consists of two neural networks: a physics discovery neural network and a predictive neural network. The former models the underlying physics of degradation, while the latter makes probabilistic predictions for degradation intensity. The physics discovery neural network guides the predictive neural network for better life estimations. The proposed framework addresses two main challenges associated with applying neural networks for lifetime estimation: incorporating the underlying physics of degradation and requirements for extensive training data. This paper demonstrates the effectiveness of the proposed approach through a case study of atmospheric corrosion of steel test samples in a marine environment. The results show the proposed framework’s effectiveness, where the mean absolute error of the predictions is lower by up to 76% compared to a standard neural network. By employing the proposed data-driven framework for lifetime prediction, systems safety and reliability can be evaluated efficiently, and maintenance activities can be optimized.

## 1. Introduction

Effective lifetime prediction for engineering systems or structures that undergo degradation in the field, such as airplanes and ships, is essential for different reasons, such as ensuring safety, facilitating preventive maintenance, and optimizing operational efficiency. For example, safety is a major concern in aviation and maritime industries. The degradation of components in a critical system/structure can lead to catastrophic failures, resulting in loss of life and significant financial implications. By accurately predicting the lifetime, potential risks can be identified in advance, allowing for proactive measures to be taken. Moreover, predicting the lifetime facilitates implementing predictive maintenance strategies, which reduces downtime and avoids costly repairs. Furthermore, accurate lifetime prediction allows for better resource allocation, improved operational planning, and enhanced efficiency.

### 1.1. Problem Statement

Two different approaches can be found in the literature for lifetime prediction: (1) empirical models based on the physics of failure and (2) data-driven models based on sensor measurements. However, both approaches have their limitations. Empirical models are obtained experimentally, usually in controlled laboratory environments. While they provide valuable insights, these models do not fully represent the actual degradation process that happens in the field. The reason is that such models have been obtained after many simplifications and may exclude certain stresses in the field (e.g., mechanical, temperature, and humidity), which can cause considerable estimation errors.

On the other hand, data-driven models, particularly neural networks (NN), can incorporate a broad range of stresses and learn the complex interactions between them. However, there are issues associated with such models as well. Firstly, they only learn the data patterns without consideration of the governing physics of degradation. Excluding physics, accompanied by the lack of interpretability in NNs, makes them prone to violating physical laws unknowingly while showing a good fit to the training data. This issue may lead to weak generalization, mainly for predicting situations that fall outside of the training dataset. Secondly, they usually require a significant amount of data for sufficient training, which may not always be available. To estimate degradation and lifetime, NNs are typically trained in a supervised setting using labeled data that ideally have been collected at different levels of degradation up to the failure points. However, collecting that data is usually expensive and time-consuming, particularly for durable systems with long lifetimes, as material degradation (e.g., corrosion, fatigue, wear, or creep) is often a slow process. Therefore, there is a need for a model that not only accounts for a wide range of stresses and captures the complex interactions between them but also should possess interpretability in the sense that it follows the underlying physics of degradation that occurs in real-world conditions. Additionally, this model should be trainable with limited data.

To address these issues, the underlying physics of degradation should be considered in developing a NN model for lifetime estimation. Incorporating the underlying physics of degradation into a NN model makes it more robust as the models’ performance does not drop for unseen data. Physics-guided models can be more accurate for predicting the state of systems in the future as physics helps determine the direction of damage accumulation. Moreover, adding physics imposes a constraint that restricts the search space for model parameters, allowing for dealing with the data scarcity issue. However, in some systems, the physics of degradation and failure is not fully known nor readily available. Therefore, the questions of how to discover the underlying physics of degradation, which may be complex in real-world conditions, and how to add the complex physics to a predictive NN for robust lifetime assessment need to be answered.

### 1.2. Previous Studies

Using NNs for lifetime prediction has been widely considered in the literature. However, previous research studies on NN models for lifetime prediction lack consideration of the common issues noted in Section 1.1. Just a limited number of studies have incorporated physics into NNs to improve their performance. A summary of examples of these studies follows.

#### 1.2.1. Neural Networks for Lifetime Prediction

NNs have found extensive applications for lifetime estimations based on sensor measurements. Batteries [1], rotating machinery [2], and machining tools [3] are some examples that have been considered in the literature for lifetime predictions by NN models. Lifetime prediction is mainly performed by estimating remaining useful life (RUL) or accumulation of damage (i.e., degradation). This estimation effort is a regression problem that maps the operating conditions to RUL or degradation intensity.

Different NN algorithms have been used for this purpose, such as feedforward NNs, convolutional NNs, recurrent NNs, and autoencoder [4,5,6]. Feedforward NNs can learn complex patterns and are relatively straightforward to implement. For example, Kang et al. [7] developed a feedforward NN for estimating RUL for turbofan engines based on sensor measurements. Khumprom et al. [8] evaluated the impact of input features on the performance of a feedforward NN for RUL prediction for the same systems. Elasha et al. [9] used the same type of network for evaluating the RUL of wind turbine gearbox bearings using some extracted features from vibration measurements. Ismail et al. trained a feedforward NN using the extracted features by principal component analysis to estimate RUL for insulated gas bipolar transistors [10].

Convolutional NNs are designed for processing grid-like data to learn hierarchical representations of input data. Liu et al. [11] trained a convolutional NN to model the RUL of bearings after a short-time Fourier transformation of collected vibrations. The same type of NN was used by Modarres et al. [12] for the assessment of structural damages. Aghazadeh et al. [3] used a convolutional NN for tool wear estimation. A convolutional NN was developed by Li et al. [13] for the RUL estimation of turbofan engines.

Recurrent NNs are designed for processing sequential data as they have internal memory to keep information from past inputs for prediction on future inputs. A long short-term memory network, which is a type of recurrent NN, was developed by Hu et al. [14] to predict the degradation of mechanical parts. Zhang et al. [15] used the same type of NN for machine tool wear prediction. Liang et al. [16] developed a recurrent NN for the life assessment of bearings.

Autoencoders can learn meaningful representations of the input data in lower dimensions and are used for feature learning. Verstraete et al. [17] and Ding et al. [18] used autoencoders for the lifetime prediction of rolling bearings. Wei et al. [19] proposed a RUL prediction framework for Lithium-ion batteries based on an autoencoder. Zhao et al. [20] compared different NN algorithms, such as autoencoder, for the lifetime prediction of machining tools.

Despite significant progress in the NN applications, to develop a robust NN model for lifetime and degradation prediction that can generalize well and deal with data scarcity, the issues of the lack of interpretability, inconsistencies with physical laws, and the requirement for extensive training data need to be addressed.

#### 1.2.2. Physics-Informed Neural Networks for Lifetime Prediction

Raissi et al. [21] introduced the idea of physics-informed neural networks (PINNs). They proposed PINN for solving challenging partial differential equations (PDE) in fluids, quantum mechanics, reaction–diffusion systems, and the propagation of non-linear shallow-water waves. PINNs are designed for supervised problems and incorporate laws of physics in the form of PDE as a penalty term to the NN cost function. This approach enables the trained model to follow the governing differential equations and be a surrogate function for the equation solution [22,23,24].

The idea of PINN was later used in a very limited number of studies for developing NN models for the prediction of degradation. Zhang et al. [25] used the idea for creep-fatigue lifetime prediction at high temperatures, while the added physics was just a simple restriction on the estimated lifetimes. They added a penalty term to the network cost function to force the lifetime estimations to be in a specific range (between 0 and 10^5^ cycles). Zhou et al. [26] added some simple fundamental rules from fatigue degradation as the underlying physics to a NN model to improve fatigue life predictions. Similarly, Kim et al. [27] considered a few basic understandings, such as the irreversibility of degradation, to guide a NN. Finally, Cofre-Martel et al. [28] used the PINN idea to explain the physics of failure based on latent features.

The major obstacle to using PINN for lifetime estimation purposes is the lack of sufficient knowledge about the underlying physics of degradation. This knowledge is often unavailable for the complex phenomena of degradation in real-world conditions, where multiple stresses act together affecting the rate and intensity of degradation synergically at different levels. That is why the previous studies mainly considered just some basic understandings of physics to guide NNs. Therefore, for lifetime prediction under degradation, there is a need for a framework that can discover the complex underlying physics of degradation and then use the discovered physics to improve the predictions of degradation.

### 1.3. Objectives and Contributions

The main objective of this paper is to introduce a NN-based approach that discovers the more sophisticated and complex underlying physics of system degradation than simplified and empirically developed relationships by considering all environmental factors that affect degradation and then use the discovered physics for a more accurate prediction of long-term degradation in the field.

For this purpose, the proposed approach creates a physics discovery NN that captures the relationship between degradation rate and operating conditions. This model is then used to guide the development of a second model, which is trained on initial measurements of degradation. The second model, known as the predictive model, learns the temporal behavior of degradation intensity while adhering to the guidance provided by the first model. By combining the physics discovery model and the predictive model, long-term degradation can be estimated accurately, even with limited training data. To enable robust decision-making, the predictive model also quantifies the uncertainty of the estimated degradation intensities, indicating the model’s confidence in its predictions.

The proposed approach has several advantages over traditional methods for predicting product degradation. Using NNs allows for more accurate predictions by identifying complex patterns and relationships in the data that may not be apparent with conventional statistical methods. The proposed NN framework incorporates underlying physics in the form of a relationship between operating conditions and degradation rate to improve the accuracy of predictions. The model also provides probabilistic estimations of accumulated damage, allowing for more robust decision-making.

The key contribution of this study is the development of a guided NN framework that incorporates the physics of degradation discovered from short-term tests to accurately predict the degradation when there is a limited knowledge about future operating conditions. Therefore, this framework effectively addresses the challenge posed by the lack of sufficient data on environmental factors that a system may encounter in the future. This challenge has been a hindrance in using machine learning models for the accurate assessment of degradation.

The approach proposed in this paper builds upon the previous research studies on using machine learning techniques to assess the life of systems experiencing environmentally induced degradation and evaluate the impact of environmental factors on degradation progress. This paper contributes to the current state of the art by introducing a novel dual NN framework. This framework involves two separate NNs working in tandem, each with distinct roles, and interacting during training to enable physics-informed predictions of degradation even when data are limited. Additionally, the implementation of this framework is demonstrated by showing the improvements in the accuracy of the predictions realized by the proposed approach. This framework enables efficient evaluation of systems degradation even with limited data, thereby avoiding the repercussions of incorrect lifetime estimations, such as economic losses and potentially life-threatening incidents, particularly for safety-critical products.

The remainder of this paper is structured as follows. The proposed framework is described in Section 2. This section explains how the framework models the underlying physics and utilizes it for degradation prediction. In Section 3, the effectiveness of the framework is evaluated for a case study on the degradation of steel coupons as a simplified form of an engineering structure. In this section, the application of the proposed approach is thoroughly evaluated, and its performance is quantified. Additionally, the obtained results are discussed. Finally, Section 4 is the conclusion. It summarizes the key findings of the study and highlights the significance of the proposed approach in predicting degradation.

## 2. Proposed Methodology

**Figure 1:**
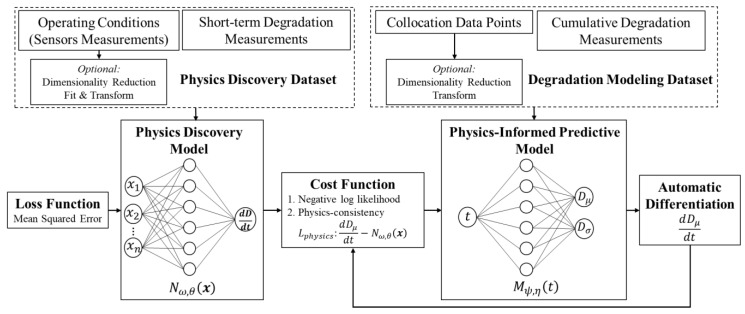
Training of guided neural network.

The proposed methodology in this paper leverages the high capacity of NNs to effectively model complex patterns and relationships within the data. By harnessing the power of NNs, the methodology aims to discover and incorporate the complex underlying physics of degradation phenomena. Figure 1 provides the overview of the training process of the proposed framework for predicting degradation intensity. The framework contains two NNs: (1) physics discovery NN ($N_{\omega ,\theta }$) and (2) predictive NN ($M_{\psi ,\eta }$). Unlike the traditional PINN, which relies on empirical models for physics, in this approach, the physics discovery NN is responsible for modeling the underlying physics of degradation. Operating conditions impact the degradation rate either linearly or non-linearly, and this linear/non-linear relationship can be captured as a regression model by the physics discovery NN. This network is a feedforward NN with mean squared error (MSE) loss function that maps the operating conditions ($x_{1}$, $x_{2}$, …, $x_{n}$) to the degradation rate ($\frac{\partial D}{\partial t}$). Equation (1) shows the MSE function, where N is the number of data points, $\frac{\partial D}{\partial t}$ is the true value of the degradation rate, and $\hat{\frac{\partial D}{\partial t}}$ is the estimated degradation rate which depends on input features x, network weights ω, and bias parameters θ.

Sensor measurements and degradation rates from short-term tests in different operating conditions are used to train the physics discovery NN. The primary goal of the physics discovery model is to capture the impact of operating conditions on the degradation rate rather than temporal patterns in degradation intensity. In this approach, it is assumed that the physics of degradation can be modeled based on the degradation rate (first-order gradient of degradation intensity with respect to time). However, if the degradation behavior undergoes significant changes over time [29], it is important to consider the degradation acceleration (second-order gradient of degradation intensity with respect to time) or higher-order gradients in modeling the underlying physics. This case can be a part of the future extensions of the current study.

The model’s ability to discover physics is reflected in its goodness of fit, indicating how effectively it captures the relationship between the variables. Additionally, the model’s capacity to discover can be further evidenced by the subsequent enhancements to the predictive model’s estimations achieved by incorporating the underlying mechanism.

(1) $$MSE=\frac{1}{N}\overset{N}{\underset{i=1}{\sum }}{\left[{\left(\frac{\partial D}{\partial t}\right)}_{i}-{\left(\frac{\hat{\partial D}}{\partial t}\left(x,\omega ,\theta \right)\right)}_{i}\right]}^{2}$$

The predictive NN estimates the degradation intensity probabilistically. The uncertainty in the estimated degradation intensity can be presented by different parametric distributions. Assuming a normal distribution with the mean of $D_{\mu }$ and standard deviation of $D_{\sigma }$, the outer layer of the predictive NN has two neurons for $D_{\mu }$ and $D_{\sigma }$. Since $D_{\sigma }$ must be a positive value, the Softplus activation function, which ensures a positive output, is used for the corresponding neuron in the outer layer. Softplus is a smooth and non-linear activation function, defined as the logarithm of the exponential function applied to the input [30]. For the other neurons, different activation functions can be used based on the ultimate performance of the model. Hyperbolic Tangent (Tanh) and Rectified Linear Unit (ReLU) are the most common activation functions for regression problems. However, some extensions of ReLU, most commonly Leaky ReLU, were also used before. Tanh may suffer from the vanishing gradient problem, which can impact training NNs.

On the other hand, ReLU avoids the vanishing gradient and is also computationally efficient. However, ReLU can cause, in some instances, some neurons to become permanently inactive. That is why the ReLU extensions were considered before. Leaky ReLU solves the inactive neuron issue, but its hyperparameter needs to be manually set, which requires additional hyperparameter tuning [30].

Equation (2) shows the cost function of the predictive NN, which has two terms: (1) $L_{data}$ for fitting the data and (2) $L_{physics}$ for following the underlying physics learned by the physics discovery NN. The model is trained using the labeled data and $L_{data}$ represents the error between the estimates (i.e., the network’s output) and the observed (true) values. $L_{physics}$ is a constraint in the optimization process of the network cost function and the scalar parameter λ weights the constraint. The proposed approach aims to achieve consistency of the predictions with the underlying physics, which is discovered by the first NN, by minimizing $L_{physics}$. The output of the predictive NN is a parametric distribution, which is why the negative log-likelihood of the actual values of degradation intensity is used as the loss term for fitting the data. In Equation (2), t is time, ψ represents the vector of weights, and η is the vector of bias parameters of the network. The automatic differentiation technique [31] is used to calculate $\frac{\partial \hat{D_{\mu }}}{\partial t}$ term in $L_{physics}$.

(2) $$Cost=L_{data}+\lambda \times L_{physics}\;L_{data}=\frac{1}{2}{\left[\frac{D-\hat{D_{\mu }}\left(t,\psi ,\eta \right)}{\hat{D_{\sigma }}\left(t,\psi ,\eta \right)}\right]}^{2}+\frac{1}{2}log{\left[\sqrt{2\pi }\times \hat{D_{\sigma }}\left(t,\psi ,\eta \right)\right]}^{2}\;L_{physics}=\frac{\partial \hat{D_{\mu }}\left(t\right)}{\partial t}-N_{\omega ,\theta }\left(x\right)$$

The physics discovery model guides the predictive model to learn the parameters so that they follow the physics alongside fitting the data. After training, only time is needed as the input for predicting field degradation intensity, as no sensor measurements are available regarding future operating conditions. It means the sensor measurements from short-term tests are used indirectly to help the predictive NN to find the right parameters for linking degradation intensity to time. So, for the prediction of degradation intensity in the future, the only available feature is use/exposure time, which is required as input.

The proposed approach uses two different datasets to train the two NNs. The first dataset, used to train the physics discovery model, has sensor measurements as input features and degradation rates as labels. However, further feature selection/extraction and data preprocessing methods can be used as required. This dataset can be obtained from short-term tests in various operating conditions. For example, samples of materials from a considered system/structure can be exposed to different environmental factors, such as temperature and humidity, within the range of its actual operating conditions in the field, and the degradation rate for each condition be measured. Using sensors, the environmental factors associated with degradation rates can be measured. The degradation rate can be quantified differently depending on the considered degradation mechanisms. For example, the degradation rate can be expressed as the resulting mass loss rate for the corrosion mechanism.

Duration and specifications of the tests vary case by case and depend on the nature of the systems and the associated failure mechanism. However, it is crucial to include critical variabilities and initial conditions that affect degradation, such as variations in geometry and environmental conditions, in the training dataset, to ensure that the physics discovery model represents and closely mimics the degradation process. To include variability in initial damages in the developed model, samples with different levels of initial degradation can be used in the short-term tests. It is worth noting that these tests are performed under operational conditions rather than accelerated conditions. The primary advantage of conducting short-term tests is the emphasis on obtaining degradation rate labels, which can be promptly collected, as opposed to measuring degradation intensity which is time-consuming for long-term degradations. The second dataset, which includes the exposure times as input features and associated degradation intensities as labels, is used for training and testing the predictive model. Similar to the first dataset, degradation intensity can be measured differently by considering the degradation mechanism. Two examples include mass loss due to corrosion and crack growth because of fatigue.

## 3. Case Study

The proposed framework is designed to predict the degradation in systems and structures when there is limited knowledge about the complex underlying physics of degradation and the impact of various operating conditions on lifetime. This case study applies the proposed framework to the steel structure of a subsystem within an aircraft working in marine environments and subject to degradation due to corrosion. However, because of the unavailability of datasets associated with the degradation of such a steel structure, degradation in coupons made from the steel is considered for this case study. Different parts of aircraft under high mechanical stresses during operation, such as landing gears, are made of steel.

In this case study we use data from the degradation of C1010 steel coupons from a previous study [32]. The C1010 is a low-carbon steel alloy known for its high weldability, ductility, and tensile strength. The coupons used in this study were exposed to atmospheric corrosion in a marine environment at the U.S. Naval Research Laboratory Key West, a testing facility situated on Fleming Key in Florida, within the U.S. Naval Air Station. The exposures were conducted between 28 August 2014 and 28 August 2015. The coupons were 3″ × 3″ × 1/6″ in dimension and were affixed with two 3/16″ diameter mounting holes facing south to ensure consistent mounting. Coupons had a glass bead blasted surface. On-site environmental data were collected from a weather station. Two datasets were gathered, one for short-term and another for long-term exposure. The link to the datasets can be found in [33]. Some specific information, such as the test standards used to prepare the coupons and the test conditions, as well as details of the instrumentation used for measurements are unknown. However, these details do not impact the results of this case study.

In this study, it is assumed that coupons represent simplified forms of a structure and degradation intensity is defined as the mass loss of the coupons resulting from corrosion over time. Mass loss was calculated as the difference between the initial mass of a coupon and its final mass after the removal of corroded mass. Thus, the initial and final mass was recorded before exposure and at the designated checkpoints for each coupon.

**Figure 2:**
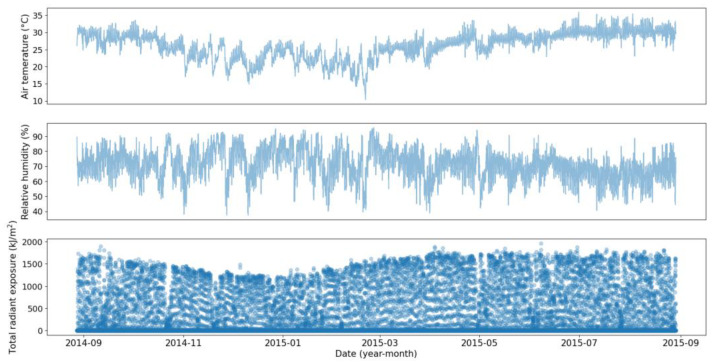
Measured operating conditions.

**Figure 3:**
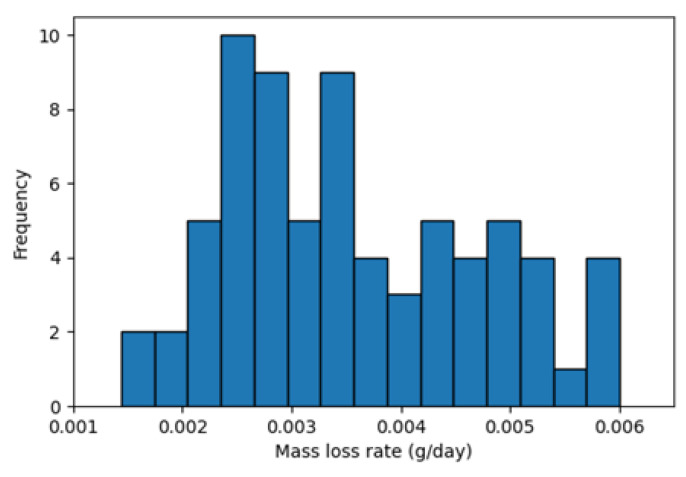
Distribution of mass loss rate (i.e., degradation rate) obtained from monthly removed coupons.

**Figure 4:**
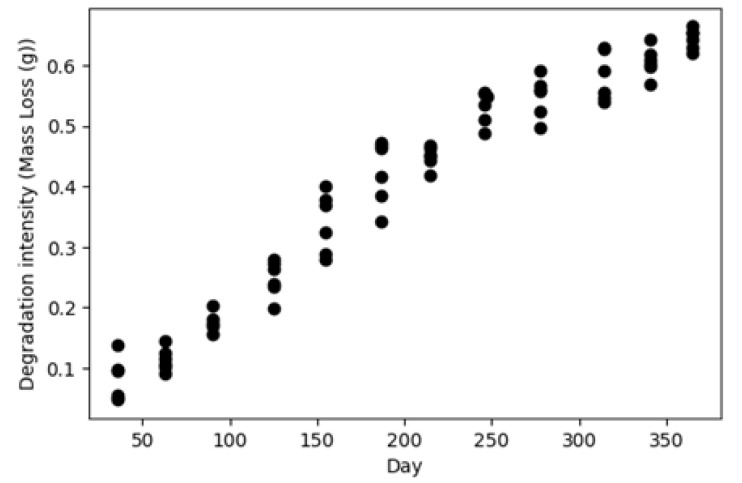
Mass loss (i.e., degradation intensity) of coupons.

The first dataset contains operating conditions and degradation measurements for 72 coupons with no initial damage which were in the field between 24 to 36 days. The operating conditions, including temperature, humidity, and solar radiation, were collected every 30 min, as shown in Figure 2, and the mass losses were recorded before removing the coupons (i.e., 72 data points). Figure 3 shows the distribution of the calculated degradation rates using this dataset. The second dataset is similar to the first dataset, except that the coupons were kept in the field for one year and the mass loss measurements were collected cumulatively at the end of each month, as shown in Figure 4.

The first dataset (i.e., short-term degradation data) is used for modeling the underlying physics by the physics discovery NN. The second dataset (i.e., cumulative degradation data for a whole year) trains and tests the physics-guided predictive NN. Different initial observations of cumulative degradations in the second dataset are used to train the predictive model, while the remaining observations are used to test the predictive model’s performance.

Twelve features, including means and standard deviations of air temperature (°C), relative humidity (%), and total radiant exposure ($\mathrm{KJ}/m^{2}$), as well as their gradients with respect to time over the month before each mass loss measurement, are used as inputs for the framework. Because of the high dimensionality of the feature space and considering the size of the dataset, a dimension reduction technique is used to extract influential features in a lower dimensional space. There are different methods for data dimension reduction, such as principal components analysis (PCA) [34], t-distributed Stochastic Neighborhood Embedding [35], and autoencoders [36] that can be used. However, to avoid the complications of data preprocessing, which is not in the scope of this paper, the straightforward linear PCA is considered. Using the PCA as an effective method for reducing the number of input features, the twelve environmental features were reduced into only two to avoid the need for a large network with many parameters. The two extracted features are linear combinations of the twelve original features, representing a signification fraction of the variance. The first two principal components, with a total variance ratio of around 0.8, retain most of the relevant information. It is worth mentioning that the first three principal components reflect a total variance ratio of above 0.9. However, using the three principal components did not improve the model performance, so the first two principal components were considered in the analysis.

A NN with a hidden layer containing four neurons is trained to model the degradation rate (i.e., mass loss rate) based on the first two principal components. The optimal size for the NN is selected based on the size of the dataset and by trial and error [37]. For larger datasets, deeper NNs can be used [38]. Tanh activation function is used in the NN as it showed desirable model performance in this case study. The goodness of fit is assessed using the mean absolute error (MAE) and the mean absolute percentage error (MAPE) of the estimates. The model produces a MAE of 0.0004 and a MAPE of 0.13, indicating a relatively good fit. The inaccuracies in the estimates may be due to the complexity of the corrosion degradation phenomenon, which depends on many conditions beyond the three conditions considered here. Therefore, it may not be possible to estimate the corrosion rate solely based on these factors accurately. Nevertheless, the model still partially learns the underlying physics relationships between the inputs and the output of the NN. This acquired knowledge can be valuable in guiding the development of a more accurate predictive model in the next step.

**Figure 5:**
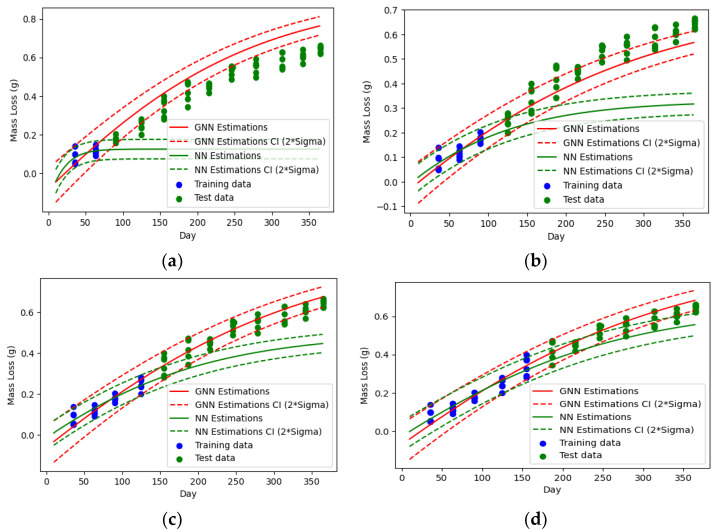
Comparing estimations of guided neural network (GNN) with regular neural network (NN) for different portions of training data: (**a**) 2 months; (**b**) 3 months; (**c**) 4 months; (**d**) 5 months.

To guide the predictive model to achieve physics-consistent estimates and limit the search space for its parameters, the physics discovery model is incorporated into the training process of the predictive model. Figure 5 compares the degradation predictions made by the guided neural network (GNN) with those made by a regular NN for different portions of training data. The dashed lines represent the distance of $2\times D_{\sigma }$ (the standard deviation of degradation intensity) above and below $D_{\mu }$ (the mean degradation intensity), which corresponds to a 95% confidence interval for the estimates. Both networks have the same structure: a one-dimensional input layer (representing time), one hidden layer of six neurons with Tanh activation functions, and two neurons at the output layer for the parameters of the probability density function of the degradation intensity ($D_{\mu }$ and $\;D_{\sigma }$) with linear and Softplus activation functions. The only difference between the two NNs is in their cost functions, where the GNN’s cost function includes a term that accounts for the physics of degradation ($L_{physics}$ in Equation (2)). A weight of $10^{5}$ is considered for the physics term ($\lambda =10^{5}$). The sensitivity analysis for the weight is provided in Appendix A.

The only input for both GNN and regular NN is time. However, the GNN is trained so that the estimated degradation intensities conform to the discovered physics (i.e., $N_{\omega ,\theta }$) during the training. For this purpose, 200 random data points (collocation points) are generated from uniform distributions within the sensor measurements range in the first dataset. These data points are then fed into the network to adjust the network parameters by forcing the model to make the automatic differentiation of the mean of the estimated distribution for degradation intensity (i.e., $\frac{\partial \hat{D_{\mu }}}{\partial t}$) close to the expected value determined by the physics discovery model (i.e., $\frac{\hat{\partial D}}{\partial t}$) for each of the collocation data points. This is achieved by minimizing the penalty term for the physics in the cost function (i.e., $L_{physics}$).

Comparing the predictions of the two NNs for the test data (i.e., the green data points in Figure 5) reveals that guiding the NN significantly improves its performance. In the case with limited training data (Figure 5a,b), the regular NN overfits the training data points and fails to learn the underlying pattern. However, the GNN provides relatively acceptable estimates for long-term degradations.

Increasing the initial observation time (i.e., the portion of training data shown by blue dots in Figure 5) results in more accurate estimations. Observing the initial degradation intensities for the first four months, the predictions of the GNN are excellent, while the regular NN shows significant errors and requires at least 5 months of training data to generate close estimations of the actual values, as demonstrated in Figure 5c,d, respectively.

**Figure 6:**
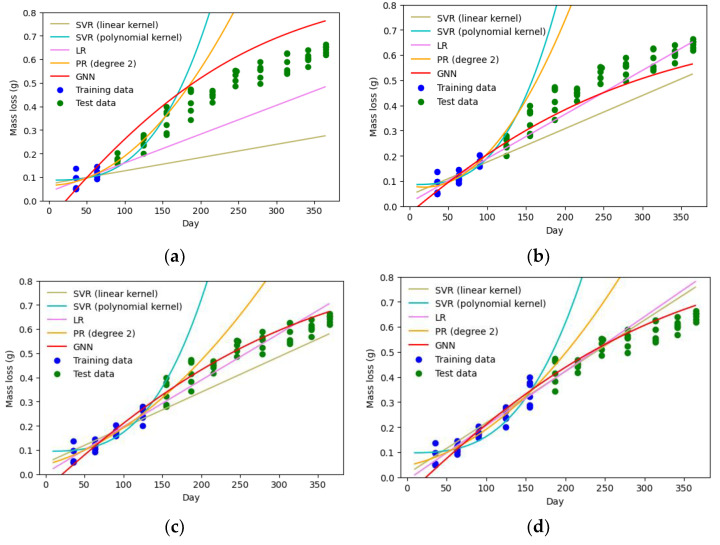
Comparing degradation prediction by different machine learning models for different portions of training data: (**a**) 2 months; (**b**) 3 months; (**c**) 4 months; (**d**) 5 months (SVR: support vector regression, LR: linear regression, PR: polynomial regression, GNN: guided neural network).

To further evaluate the proposed method’s performance, its prediction accuracy is compared to other machine learning techniques, including support vector regression (SVR), linear regression (LR), and polynomial regression (PR) (see Table 1). Non-linear models of PR and SVR with polynomial kernel function show the worst performances, as shown in Figure 6. Although they represent a good fit for the training data, their estimates for the test data points are considerably inaccurate.

Linear models, including LR and SVR with linear kernel, perform better than the non-linear models. However, they cannot consistently predict the degradation intensities with accuracy. This is because the degradation phenomena have a highly non-linear nature, which makes the linear models perform well only on certain parts of the period (e.g., the middle portion in Figure 6d). In addition, when the training data is limited, their estimates differ significantly from the actual values, as shown in Figure 6a.

Table 2 presents the goodness of fit to the test data for the considered models with different observation times (i.e., training data), allowing for a comparison of their performance in predicting the degradation intensity. Four evaluation metrics are used: MAE, maximum error (ME), MAPE, and $R^{2}$, which are calculated using Equations (3)–(6), where D is actual degradation intensity, $\hat{D}$ is estimated degradation intensity, N is the number of data points, $\bar{D}$ is the mean of the actual values of degradation intensity, and ν is a small positive number to avoid undefined function when D is zero.

(3) $$\mathrm{MAE}\left(D,\hat{D}\right)=\frac{1}{N}\overset{N}{\underset{i=1}{\sum }}\left|D_{i}-{\hat{D}}_{i}\right|$$

(4) $$\mathrm{ME}\left(D,\hat{D}\right)=max\left(\left|D_{i}-{\hat{D}}_{i}\right|\right)$$

(5) $$\mathrm{MAPE}\left(D,\hat{D}\right)=\frac{1}{N}\overset{N}{\underset{i=1}{\sum }}\frac{\left|D_{i}-{\hat{D}}_{i}\right|}{max\left(\nu ,\left|D_{i}\right|\right)}$$

(6) $$R^{2}\left(D,\hat{D}\right)=1-\frac{\sum_{i=1}^{N}{\left(D_{i}-{\hat{D}}_{i}\right)}^{2}}{\sum_{i=1}^{N}{\left(D_{i}-{\bar{D}}_{i}\right)}^{2}}$$

Among the considered models, GNN exhibits the best performance, although for one of the cases (3 months of observation) LR shows a slightly better fit based on three of the parameters (MAE, MAPE, and $R^{2}$). However, the ME of the GNN is still lower than the LR model, which means even for this specific case LR estimations are worse than GNN for some parts of the period. It should be noted that LR is a linear model and not suitable for modeling physical degradation progress due to its disability to capture non-linearity. The good performance of LR observed for some cases (e.g., 3 months and 4 months of observation cases in Table 2) is likely due to the low level of non-linearity of the cases considered for the specific period (1 year). However, collecting degradation data over a longer time (more than a year) would likely result in a higher non-linearity and can underline the weakness of LR more clearly. On the other hand, GNN’s ability to learn non-linear patterns in the data is evident from Figure 6.

The models considered in this study exhibit good fits to the training data for all cases, except for the 2-month observation data, as shown in Table 3. This indicates the potential for overfitting for the models with low performance on the test data. GNN exhibits the worst fit for the training data yet performs the best on the test data. This suggests that the GNN model is not overfitting the training data. In contrast, the other models exhibit lower performance on the test data compared to their performance on the training data, indicating overfitting.

Finally, although in this case study the performance of the GNN on test and training data is evaluated to demonstrate the proposed approach capability for predicting degradation intensity, there are other approaches, such as the formal methods described in [39,40] that can be employed to validate the model. Considering these approaches can benefit future extensions of this study.

## 4. Conclusions

Machine learning models used for degradation prediction are purely data-driven approaches and may not accurately predict degradation when insufficient data exist. Furthermore, they do not consider the underlying physics of degradation. This paper proposes a GNN framework for incorporating complex physics to facilitate physics-consistent prediction of field degradation with limited data. The proposed method is a dual NN framework consisting of two NNs for discovering the underlying physics and predicting degradation intensity. The physics discovery NN relies on environmental factors such as temperature and humidity affecting the degradation rate. Therefore, the underlying physics can be discovered by modeling the degradation rate based on the data obtained from short-term tests and be added as a penalty term to the cost function of the predictive NN for estimating degradation intensity. The predictive model can be trained with limited data by respecting the discovered physics as a constraint that limits the search space for the model parameters. Further, fitting the data and following the physics simultaneously makes the model generalize well for unseen field data.

The performance of the proposed framework is evaluated through a case study of the atmospheric corrosion of steel coupons as simplified forms of a structure. The results indicate the potential of the proposed GNN for predicting long-term degradations based on limited observations of the initial degradation intensities. At the same time, other machine learning models may overfit the data. The proposed approach enables the efficient evaluation of the lifetime of systems and structures, thereby ensuring their safety and reliability as well as minimizing their maintenance costs. Accurate lifetime estimation allows for appropriate risk management measures to prevent accidents and ensure reliable functionality. Additionally, optimized predictive maintenance can be implemented to avoid unnecessary repairs and unexpected downtimes.

Finally, it is important to acknowledge the limitations of the proposed framework. Firstly, the framework requires degradation data for training purposes, albeit less data than a regular NN. However, collecting such data can be time-consuming and expensive as degradation is often a slow process. Moreover, the framework primarily focuses on degradation. However, potential failures may occur in the field due to discrete over-stress conditions rather than gradual and temporal degradation. Lastly, systems may involve high variations and uncertainties in their degradation behaviors in real-world situations, depending on their operating conditions. These variations may exist even in identical systems. This paper has not considered these variations and the resulting uncertainties in the degradation predictions. Such assessments can be viewed as future extensions of this work.

## Figures and Tables

**Table 1:** Considered models for comparison with the guided neural network (GNN).

#	Model	Specifications
1	Support vector regression	Linear kernel, C = 0.1, ε = 0.05
2	Support vector regression	Polynomial kernel, degree 2, C = 0.1, ε = 0.05
3	Linear regression	Least squares method
4	Polynomial regression	Degree 2, Least squares method

**Table 2:** Metrics for quantifying the quality of degradation predictions (test data).

Training Data	Model	Mean Absolute Error	Max Error	Mean Absolute Percentage Error	$R^{2}\mathrm{Score}$
2 months	SVR (linear)	0.257	0.387	0.535	−2.450
	SVR (poly)	0.995	3.100	1.725	−93.357
	LR	0.135	0.215	0.285	0.030
	PR	0.383	1.094	0.681	−11.801
	Regular NN	0.335	0.539	0.683	−5.042
	GNN	0.095	0.170	0.227	0.520
3 months	SVR (linear)	0.115	0.185	0.236	0.05
	SVR (poly)	1.667	4.553	2.902	−315.067
	LR	0.047	0.130	0.102	0.776
	PR	0.767	1.871	1.372	−58.94
	Regular NN	0.206	0.347	0.394	−2.157
	GNN	0.059	0.109	0.119	0.723
4 months	SVR (linear)	0.084	0.152	0.166	0.163
	SVR (poly)	1.303	3.287	2.242	−284.497
	LR	0.048	0.108	0.095	0.697
	PR	0.238	0.589	0.417	−8.231
	Regular NN	0.134	0.217	0.249	−1.042
	GNN	0.032	0.075	0.068	0.849
5 months	SVR (linear)	0.060	0.142	0.107	0.136
	SVR (poly)	1.149	2.626	1.958	−324.905
	LR	0.069	0.162	0.123	−0.166
	PR	0.335	0.726	0.578	−25.327
	Regular NN	0.064	0.108	0.116	0.199
	GNN	0.036	0.088	0.068	0.704

**Table 3:** Metrics for quantifying the quality of fitting degradation data (training data).

Training Data	Model	Mean Absolute Error	Max Error	Mean Absolute Percentage Error	$R^{2}\mathrm{Score}$
2 months	SVR (linear)	0.022	**0.047**	0.291	0.200
	SVR (poly)	0.022	**0.047**	0.291	0.200
	LR	0.022	0.057	0.270	0.283
	PR	0.022	0.057	0.270	0.283
	Regular NN	0.022	0.057	0.269	0.283
	GNN	0.0307	0.089	0.289	−0.655
3 months	SVR (linear)	0.022	0.047	0.252	0.657
	SVR (poly)	0.020	0.047	0.233	0.681
	LR	0.020	0.061	0.213	0.705
	PR	0.018	0.057	0.210	0.729
	Regular NN	0.020	0.062	0.220	0.688
	GNN	0.021	0.076	0.195	0.632
4 months	SVR (linear)	0.025	0.050	0.244	0.808
	SVR (poly)	0.023	0.050	0.221	0.831
	LR	0.021	0.064	0.181	0.864
	PR	0.020	0.059	0.183	0.871
	Regular NN	0.021	0.067	0.185	0.853
	GNN	0.024	0.098	0.191	0.757
5 months	SVR (linear)	0.029	0.070	0.219	0.875
	SVR (poly)	0.030	0.061	0.219	0.871
	LR	0.025	0.074	0.166	0.893
	PR	0.025	0.061	0.172	0.907
	Regular NN	0.027	0.081	0.172	0.881
	GNN	0.029	0.103	0.195	0.849

**Figure A1:**
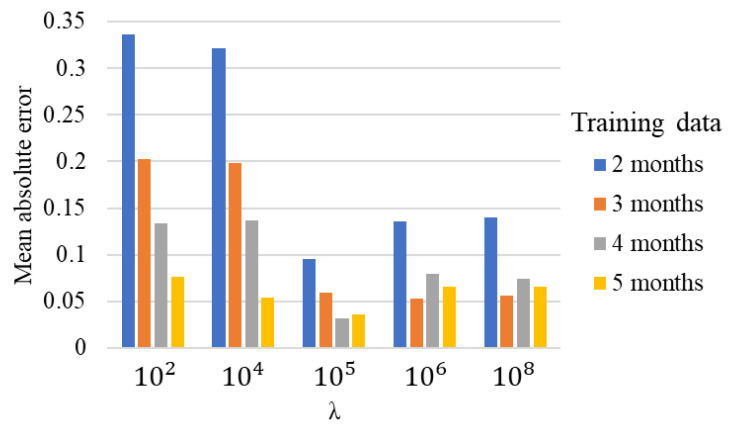
Sensitivity analysis of physics term weight λ for test dataset with different portions of training data.

## Data Availability

Data is publicly available at https://osf.io/sgeun/ (accessed on 22 January 2023).

## Appendix A

Figure A1 presents the sensitivity analysis results for the physics term weight λ. For lower values of λ ($10^{2}$ and $10^{4}$), the model prioritizes fitting the training data over following the physics. So, it overfits the training data, which is why the prediction error for unseen test data is relatively high. For higher values of λ ($10^{5}$, $10^{6}$, and $10^{8}$), the prediction error drops since increasing λ value improves the model’s generalization ability as it considers both fitting the training data and adhering to the physics. This leads to a more accurate prediction. However, when λ value exceeds $10^{5}$, the model adheres more to the physics and gives less consideration to fitting the data, resulting in a minor increase in prediction error.
